# Hybrid artificial intelligence and quantum annealing as an optimization layer in drug discovery

**DOI:** 10.1016/j.patter.2026.101634

**Published:** 2026-08-05

**Authors:** Chia-Ho Ou, Jun-Cheng Liao, Chung-Yao Huang, Oscar K. Lee

**Affiliations:** 1 Graduate School of Information Sciences, Tohoku University, Sendai, Japan; 2 Department of Computer Science and Information Engineering, National Pingtung University, Pingtung, Taiwan; 3 Department of Biotechnology Medicine, MacKay Memorial Hospital, Taipei, Taiwan; 4 AI Application Department, MacKay Memorial Hospital, Taipei, Taiwan

**Keywords:** AI-driven drug discovery, QUBO optimization, quantum annealing, hybrid computational workflows, multi-objective optimization

## Abstract

Artificial intelligence (AI) has greatly expanded the generative capacity of drug discovery, yet the ability to translate large candidate pools into structured, resource-aware decisions remains limited. This review addresses this emerging optimization bottleneck by examining quantum annealing as a potential decision-optimization layer within hybrid computational workflows. We focus on discrete, constraint-dominated tasks—such as compound subset selection, combinatorial design, and multi-objective prioritization—that can be formulated as quadratic unconstrained binary optimization (QUBO) problems. Rather than positioning quantum annealing as a predictive tool, we analyze its role as a complementary optimization interface integrated with AI-generated scores. We further discuss practical implementation considerations, including embedding overhead, noise, scalability, and benchmarking challenges. By emphasizing workflow-level design and rigorous evaluation, this review provides a pragmatic framework for assessing annealing-based optimization in drug discovery and related data-intensive scientific domains.

## The bigger picture

Artificial intelligence (AI) has captured widespread attention for its ability to rapidly design and propose thousands of potential new drug candidates. This massive computational expansion, however, has created an unexpected downstream challenge: generating candidate molecules is no longer the main bottleneck in drug discovery. The true bottleneck is deciding which ones to actually manufacture and test under strict real-world constraints. Choosing the best subset of molecules requires balancing competing goals, such as high effectiveness, low toxicity, chemical diversity, and tight experimental budgets. This review explores how a specialized computing approach called “quantum annealing” can help solve this decision-making challenge. Unlike predictive AI models that learn from data to guess molecular properties, a quantum annealer acts strictly as a dedicated problem solver. It maps complex, real-world choices into an energy landscape where different combinations of decisions represent different energy levels. By utilizing the physics of quantum mechanics, the system naturally searches this landscape to find the lowest-energy state, which corresponds to the most optimal, cost-effective decision strategy.

Ultimately, this challenge extends far beyond medicine. Across data-driven fields such as materials science and synthetic biology, scientists are discovering that as generative AI expands what is possible, selecting the right options becomes a critical systems barrier. Developing flexible architectures that couple generative intelligence with hardware-aware optimization provides a generalizable framework for data-intensive science. By establishing a rigorous evaluation process that compares these methods against classical benchmarks, researchers can move past the hype to deploy alternative computing strategies where they are genuinely needed most.

## Graphical abstract

**Figure:**
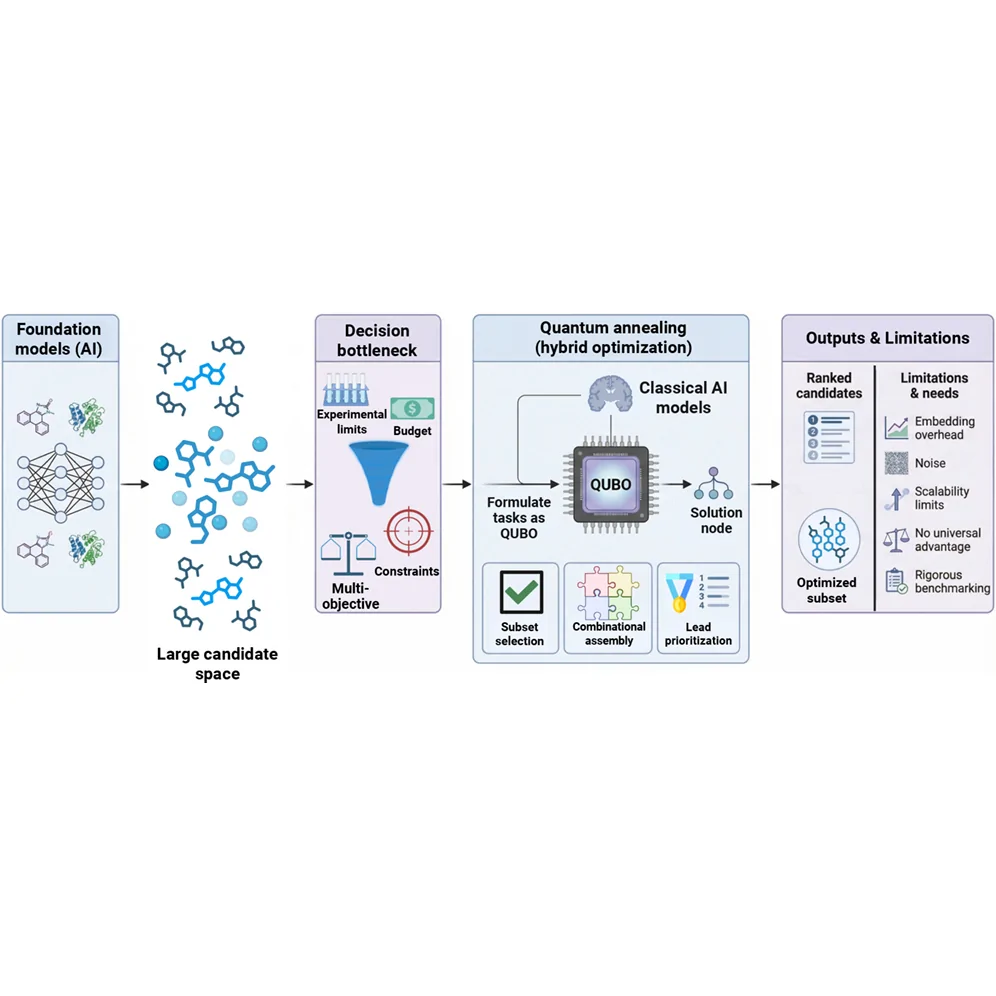

## Teaser

Artificial intelligence has significantly expanded candidate generation in drug discovery, but selecting optimal compounds under real-world experimental constraints remains a major bottleneck. This review examines quantum annealing as a potential downstream optimization layer within hybrid computational workflows. By focusing on constraint-dominated decision tasks formulated as quadratic unconstrained binary optimization (QUBO) problems, the authors analyze how annealing architectures complement AI-generated scores. They further discuss practical hardware limitations, reporting considerations, and the critical role of rigorous benchmarking against classical baselines.

## Introduction

Artificial intelligence (AI) has rapidly become a foundational technology in modern drug discovery, reshaping multiple stages of the pipeline from target identification to lead generation.^1^^,^^2^^,^^3^^,^^4^ Advances in deep learning, representation learning, and large-scale data integration allow models to extract complex patterns from chemical and biological datasets that were previously difficult to analyze using conventional computational approaches.^5^^,^^6^ In particular, the emergence of foundation models for proteins and small molecules has substantially expanded the accessible design space, enabling rapid generation of diverse candidate structures with potential biological relevance.^7^^,^^8^^,^^9^

However, this expansion of generative capacity has revealed an important downstream bottleneck. Although AI models can propose large numbers of candidate compounds or sequences, translating this abundance into viable drug candidates remains challenging.^2^^,^^10^ Drug discovery is fundamentally a multi-objective decision process that must simultaneously consider potency, selectivity, pharmacokinetics, safety, manufacturability, and other developability constraints.^11^ These objectives are often competing, nonlinear, and context dependent, and many are only partially captured by predictive models. As a result, the central challenge in contemporary AI-driven drug discovery is increasingly not how to generate candidates but how to select and prioritize them under realistic constraints.

Classical optimization approaches such as genetic algorithms, simulated annealing, and Bayesian optimization remain widely used to address these problems.^12^^,^^13^^,^^14^^,^^15^^,^^16^ These methods have proven effective in many settings, including molecular property optimization and protein design. Nevertheless, their performance can degrade as the dimensionality of the design space increases and constraints become more numerous or discrete. Many drug-discovery tasks involve combinatorial explosions arising from binary or categorical choices, such as fragment inclusion, residue substitution, or compound subset selection, which are difficult to explore efficiently using classical heuristics alone.

These challenges have motivated interest in complementary optimization paradigms. One such approach is quantum annealing (QA), which focuses on solving discrete optimization problems formulated as quadratic unconstrained binary optimization (QUBO) models or equivalent Ising formulations.^17^^,^^18^^,^^19^^,^^20^ Unlike predictive AI models, QA does not infer molecular properties or biological mechanisms.^21^ Instead, it operates as an optimization layer that can incorporate scores generated by AI models or simulations while explicitly encoding constraints within the objective function. In this way, annealing-based approaches may assist with structured decision-making in complex design spaces.

Broader quantum-computing approaches in healthcare and drug discovery include gate-based algorithms for quantum chemistry, quantum machine learning, cryptography/security, and optimization-oriented paradigms such as QA. Recent reviews have emphasized that these approaches differ substantially in hardware requirements, maturity, and near-term plausibility.^22^^,^^23^^,^^24^ This review therefore deliberately narrows its scope: we focus on QA because it provides a directly usable optimization interface for QUBO/Ising formulations while treating it as one component within the broader landscape of quantum computing for biomedical and scientific decision-making.

In this review, we examine how QA may complement AI-enabled drug-discovery pipelines by addressing downstream optimization problems. We first summarize the role of AI foundation models in contemporary discovery workflows and the optimization challenges they create. We then discuss how drug-discovery decision tasks can be formulated as QUBO problems and integrated into hybrid computational workflows. Throughout, we emphasize realistic use cases, practical limitations, and the importance of rigorous benchmarking when evaluating emerging optimization technologies.^25^

## AI foundation models in contemporary drug discovery

AI applications in drug discovery have evolved from task-specific predictive models toward large foundation models capable of learning transferable representations across diverse biological and chemical contexts. These models are typically trained on large-scale molecular, protein, and biological datasets and can be adapted to multiple downstream tasks with limited additional supervision, enabling unified representations of molecules, proteins, and biological systems. Large-scale curated chemical and bioactivity databases, such as ChEMBL, have provided important data resources for training, benchmarking, and evaluating molecular prediction and generative models.^26^

### Protein foundation models

Protein foundation models are typically trained on millions or billions of amino acid sequences using self-supervised learning strategies adapted from natural language processing. By treating sequences as biological “sentences,” these models learn embeddings that capture structural, functional, and evolutionary information.^27^^,^^28^^,^^29^ Such representations have been successfully applied to tasks including structure prediction, mutational effect prediction, and protein design.

The development of large-scale protein language models and structure prediction methods has dramatically expanded the availability of structural and functional information about proteins.^27^^,^^28^ These advances have enabled rapid *in silico* exploration of sequence variants and functional motifs relevant to enzyme engineering and therapeutic protein design. Nevertheless, protein foundation models remain limited in their ability to capture dynamic conformational changes, cellular context, and higher-order biological regulation, meaning that their predictions often require integration with additional modeling approaches or experimental validation.^30^

### Molecular generative models

Parallel advances have occurred in the modeling of small molecules. Modern generative models represent molecules using graphs, sequences such as SMILES strings, or three-dimensional structures and employ architectures including transformers, graph neural networks, and diffusion models.^31^^,^^32^^,^^33^ These models can generate novel compounds conditioned on desired properties, enabling *de novo* molecular design rather than reliance on predefined chemical libraries.

Diffusion-based and autoregressive generative models have shown particular promise in producing chemically valid and diverse molecules and exploring new regions of chemical space.^32^^,^^34^^,^^35^ In medicinal chemistry workflows, they are increasingly used to generate analog series, explore local chemical neighborhoods, and propose scaffold modifications around known hits. However, while generative models expand the range of possible candidates, they do not inherently solve the problem of selecting optimal molecules under multiple constraints. Reinforcement learning or conditional generation can partially guide this process, but their effectiveness decreases as the number and complexity of objectives increases.^36^

### Multimodal foundation models

Recent work has extended foundation models toward multimodal architectures that integrate heterogeneous biological data, including molecular structure, gene expression profiles, phenotypic screening data, and clinical information.^37^^,^^38^ These approaches aim to capture relationships across biological scales and improve predictions of drug-target-disease interactions. Despite their promise, multimodal models introduce additional challenges related to data integration, interpretability, and computational cost. Aligning heterogeneous datasets often requires simplifying assumptions or proxy labels, and model performance may degrade when extrapolating beyond the training distribution.^39^ As model complexity increases, understanding uncertainty and failure modes becomes increasingly important for decision-making in drug discovery.^40^

## The persistent optimization gap

Across protein, molecular, and multimodal foundation models, a recurring theme is the rapid expansion of candidate generation capacity without a corresponding improvement in downstream optimization. AI models are highly effective at scoring or sampling candidate molecules, but drug discovery ultimately requires discrete decisions about which compounds to synthesize, which variants to test, and which leads to advance. These decisions must consider multiple objectives and constraints that cannot easily be reduced to a single scalar score.^11^ As the size of candidate pools grows, selecting optimal subsets becomes increasingly difficult. This gap between generative capacity and decision-making efficiency motivates the exploration of complementary computational frameworks that focus on structured optimization rather than representation learning alone.

### Optimization challenges in AI-driven drug discovery

Drug discovery is inherently an optimization problem characterized by discrete decisions, competing objectives, and incomplete information. As AI models generate larger and more diverse candidate sets, the challenge of selecting and prioritizing candidates under realistic constraints has become increasingly pronounced.

### Multi-objective optimization and constraints

Drug discovery requires balancing multiple objectives, including potency, selectivity, pharmacokinetics, toxicity, and manufacturability.^11^^,^^41^ Improvements in one property frequently come at the expense of another, making multi-objective optimization unavoidable. While predictive models can estimate individual properties, integrating these predictions into coherent decision strategies remains difficult.

Constraints further complicate the problem. Medicinal chemistry rules, synthetic feasibility, intellectual property considerations, and regulatory requirements impose boundaries on acceptable solutions.^42^ Many of these constraints are discrete, requiring binary decisions such as inclusion or exclusion of specific chemical fragments or sequence variants.

### Combinatorial search spaces

Many downstream drug-discovery tasks involve combinatorial decision spaces. Selecting subsets of compounds, assembling fragments, or choosing residue substitutions all involve discrete choices whose combinations grow exponentially with problem size.^43^^,^^44^ Even relatively modest design problems can therefore generate extremely large search spaces that are impractical to explore exhaustively.

AI models often exacerbate this challenge by increasing the number of plausible candidates rather than reducing it. While this diversity supports exploration, it places additional pressure on optimization methods to identify promising candidates efficiently.

### Toward new optimization paradigms

These challenges highlight an emerging optimization gap in AI-driven drug discovery. While representation learning and generative modeling have advanced rapidly, methods for structured decision-making under constraints have evolved more slowly. As a result, growing attention has focused on alternative optimization paradigms that can naturally represent binary variables, explicitly encode constraints, and explore large combinatorial spaces. QA represents one such approach. By formulating optimization problems as energy minimization over binary variables, annealing-based methods provide a framework for addressing discrete decision problems common in drug discovery.^17^^,^^18^ Rather than replacing AI models, they may serve as complementary tools for candidate selection and prioritization within hybrid computational pipelines (Table 1).

**Table 1:** Comparison of optimization paradigms in AI-driven drug discovery

Method	Handles discrete variables	Multi-objective optimization	Scalability in high dimensions	Explicit constraint encoding	Hardware dependency	Typical use cases
Genetic algorithms	yes	yes	moderate (population-based scaling)	implicit (via fitness penalties)	no	molecular evolution, sequence design
Simulated annealing	yes	limited (scalar objective)	moderate	implicit (penalty-based)	no	local search, conformational sampling
Bayesian optimization	limited	limited (typically scalarized)	poor in very high dimensions	weak (soft constraints)	no	expensive function optimization
Reinforcement learning	limited	yes (reward shaping)	variable (data dependent)	implicit (reward design)	no	generative molecule optimization
Digital annealers/quantum inspired	yes	yes	high (problem dependent)	explicit (QUBO/Ising)	specialized classical hardware	portfolio optimization, subset selection
Quantum annealing	yes	yes (via energy formulation)	limited by embedding and qubit count	explicit (QUBO penalties)	quantum hardware	combinatorial subset selection, constrained decision optimization

## QA in practice: State-of-the-art hardware, software stack, and implications for drug discovery

QA is most usefully evaluated not as a general-purpose quantum computer but as a hardware-constrained optimization substrate that samples low-energy solutions of Ising/QUBO models under real-world limitations: sparse connectivity, analog control errors, finite temperature, and calibration-dependent variability. These features shape what problem sizes are practically solvable, how constraints should be encoded, and how results should be benchmarked in drug-discovery pipelines. In this section, we review the current state of the art in QA hardware (with a focus on the D-Wave platform), explain how hardware constraints translate into modeling overhead (minor embedding and chained variables), and summarize workflow patterns most relevant to AI-enabled drug discovery.

### Current state-of-the-art QA hardware: The D-Wave platform

#### Native connectivity and processor topologies

Modern QA processors implement a native hardware graph rather than an all-to-all connectivity scheme. D-Wave’s generations are commonly discussed in terms of their topologies: Chimera (earlier systems), Pegasus (Advantage generation), and Zephyr (Advantage2 generation). Connectivity is often summarized by qubit degree, which has increased over generations, e.g., Chimera degree 6 vs. Pegasus degree 15 vs. Zephyr degree 20, improving the efficiency of certain embeddings and enabling denser native substructures.^45^^,^^46^^,^^47^

From a workflow perspective, increased connectivity reduces (but does not eliminate) the need to “simulate” missing couplers through embedding. Even on modern devices, most drug-discovery decision problems (e.g., dense similarity penalties, coverage/diversity interactions, and multi-constraint portfolios) do not directly match the native topology, so a mapping step is still required.

#### From logical variables to physical qubits: Minor-embedding and chained variables

When a problem’s interaction graph is not a subgraph of the hardware topology, it must be mapped via minor embedding, in which a single logical variable is represented by a chain of physical qubits coupled strongly so that they behave as one variable.^48^ This mapping has direct consequences for scalability and reproducibility in practice. First, the number of physical qubits required can increase substantially relative to the number of logical decision variables, meaning the effective problem size that can be realized on hardware is often much smaller than what is implied by nominal qubit counts. Second, the embedded representation introduces an additional modeling parameter, chain strength, that must be tuned: if chains are too weak, chain breaks can occur and degrade solution quality; if chains are too strong, the intended energy landscape can be distorted and useful dynamics suppressed. Third, because the usable “working graph” and calibration quality can vary across hardware updates, the same logical formulation may embed differently or perform differently over time, reinforcing the need to report embedding statistics and to rely on repeated sampling and stability analysis when drawing conclusions.

#### Noise, analog control errors, and reproducibility

QA hardware operates as an analog device and is subject to noise and control errors. Even when a QUBO is fixed, sampled solutions can vary across runs, and results are sensitive to parameter choices (anneal schedule/time, gauge transforms, number of reads, chain strength, and post-processing). Modern software tooling (e.g., virtual graph workflows) partially mitigates some systematic biases and helps reuse embeddings across runs but does not remove the need for repeated sampling, aggregation, and stability analysis in scientific benchmarking. These device-level sources of variability motivate a hardware-aware reporting practice to ensure that results can be interpreted, reproduced, and compared fairly across studies.^48^

### Hybrid solvers in current practice: Scaling beyond direct embedding

In current practice, many problem instances of practical interest exceed what can be directly embedded on a quantum processing unit (QPU) once connectivity constraints and chain overhead are accounted for. As a result, hybrid quantum-classical approaches have become a common route for addressing larger or denser formulations: classical routines decompose the original optimization task into subproblems that fit the hardware constraints, the QPU is used to sample low-energy solutions for selected subproblems, and classical coordination steps reconcile partial solutions and enforce global feasibility. This workflow shifts the evaluation focus from whether a single QPU call outperforms a classical heuristic in isolation to whether the end-to-end hybrid pipeline improves decision quality, robustness, or time to target under realistic constraints.

Importantly, improvements in hardware connectivity (e.g., moving from earlier topologies to Pegasus/Zephyr-family designs) can reduce embedding overhead for certain graph structures. Still, they do not eliminate the need for hybridization when objectives involve dense interactions or when constraints introduce auxiliary variables that expand the effective problem size. Therefore, when positioning QA within drug-discovery workflows, it is most informative to describe how hybrid strategies are used in practice (problem decomposition, sampling protocol, and post-processing) and to benchmark them against well-tuned classical baselines using decision-relevant metrics rather than energy values alone.

### QUBO/Ising as the interface between drug-discovery decisions and QA hardware

The practical interface between QA and real-world problems is the Ising/QUBO formulation: minimizing an energy function over binary variables. In drug discovery, this interface is most relevant for downstream decision problems in which AI/simulation/assays generate and score candidates, and QA optimizes selection and configuration under constraints.

In QUBO form, each decision is encoded as a binary variable (e.g., select/exclude a compound, include/exclude a fragment, or choose a residue option), linear terms reflect external scores, and quadratic terms encode interactions such as redundancy penalties, diversity constraints, or mutual exclusivity. Constraints are typically enforced via penalty terms. In practice, inequality constraints and higher-order interactions often require auxiliary variables and quadratization, increasing problem size and embedding overhead, an issue that becomes central once hardware constraints are considered. A concise, drug-discovery-oriented explanation is provided in Boxes 1 and 2.

Box 1 **Quantum annealing and QUBO explained for drug-discovery scientists**

Quantum annealing is an optimization approach used to solve complex decision-making problems rather than a method for predicting molecular properties. In drug-discovery applications, it is best understood as a tool for selecting optimal combinations of candidates or experimental choices under multiple objectives and constraints.

To apply quantum annealing, a problem is formulated as a QUBO model. In a QUBO formulation, each decision is represented by a binary variable, such as whether to include or exclude a specific compound from a candidate set. An objective function defines an overall “energy” that combines desirable scores (e.g., predicted potency, selectivity, or safety) with penalty terms that enforce practical constraints, such as budget limits, chemical-feasibility rules, or portfolio-balance requirements.

The quantum annealer searches for binary decision combinations that minimize this energy, where lower energy corresponds to a better overall decision according to the specified criteria. Importantly, quantum annealing does not generate molecules, simulate chemical interactions, or replace predictive models. Instead, it complements classical machine learning and simulation methods by optimizing downstream decisions based on their outputs.

Box 2 **Formal QUBO formulation for compound subset selection with diversity and budget constraints**

To make the QUBO mapping explicit, consider a candidate set of *N* molecules and define binary variables *x*_*i*_ ∈ {0,1}, where *x*_*i*_ = 1 indicates that compound *i* is selected for synthesis or experimental testing. Let *a*_*i*_ denote a normalized desirability score, such as predicted potency adjusted for toxicity, and let *S*_*ij*_ ∈ [0,1] denote a pairwise similarity or redundancy score between compounds *i* and *j*. Let *c*_*i*_ denote the experimental cost of testing compound *i*, and let *B* be the available budget after costs have been scaled to integer units. If an assay campaign is designed to select exactly *K* compounds while respecting the true upper-bound budget constraint $\sum_{i}c_{i}x_{i}\leq B$, the optimization objective can be written as a QUBO by introducing binary slack variables *y*_*m*_ that represent unused budget:

$$E\left(x,y\right)=-\alpha \sum_{i}a_{i}x_{i}+\beta \sum_{i<j}S_{ij}x_{i}x_{j}+\lambda_{K}{\left(\sum_{i}x_{i}-K\right)}^{2}+\lambda_{B}{\left(\sum_{i}c_{i}x_{i}+\sum_{m}2^{m}y_{m}-B\right)}^{2}\text{.}$$

Here, *α*,*β*,*λ*_*K*_,*λ*_*B*_ > 0. The first term rewards high-scoring candidates, the second penalizes co-selection of highly similar compounds to encourage diversity, the third enforces the desired subset size, and the fourth enforces the budget as a true inequality constraint. The slack term represents unused budget, so a feasible solution with $\sum_{i}c_{i}x_{i}\leq B$ can achieve zero budget penalty when the slack variables satisfy $\sum_{m}2^{m}y_{m}=B-\sum_{i}c_{i}x_{i}$. The number of slack bits should be chosen so that all feasible unused-budget values can be represented after integer scaling.

Under the standard upper-triangular QUBO convention, the same objective can be written as

$$E\left(z\right)=\sum_{p}Q_{pp}z_{p}+\sum_{p<q}Q_{pq}z_{p}z_{q}+\text{constant.}$$

Here, *z* collects all binary variables in the formulation, including the compound-selection variables *x*_*i*_ and the slack variables *y*_*m*_. The index *p* therefore refers to any one binary variable in this combined vector. The diagonal coefficient *Q*_*pp*_ is the net linear weight associated with variable *z*_*p*_; for binary variables, this contribution is placed on the diagonal because $z_{p}^{2}=z_{p}$. The off-diagonal coefficient *Q*_*pq*_, with *p* < *q*, is the quadratic coupling between variables *z*_*p*_ and *z*_*q*_. In this example, such couplings arise from the pairwise similarity penalty, the fixed-cardinality penalty, and the slack-variable budget penalty. Thus, *Q*_*pp*_ and *Q*_*pq*_ are not additional biological inputs; they are obtained by expanding the objective function and collecting the resulting linear and quadratic terms. This compactly maps AI-derived utility scores, diversity penalties, subset-size control, and true budget inequalities into a QUBO objective without treating the budget as an equality constraint.

The introduction of slack variables makes the inequality budget constraint compatible with QUBO solvers, but it also increases the number of binary variables and couplers. This directly increases embedding overhead on quantum annealing hardware and may increase the complexity of hybrid decomposition. Penalty weights such as *λ*_*K*_ and *λ*_*B*_ must therefore be large enough to discourage infeasible solutions but not so large that they compress the desirability and diversity terms or degrade analog precision. In practice, penalty tuning is part of the modeling workflow rather than a purely technical afterthought.

This example also clarifies the role of quantum annealing as a downstream decision-optimization interface rather than a predictive model. AI models, docking, simulations, or experimental data provide the utility scores, cost estimates, constraints, and pairwise relationships; the annealing-based optimizer searches for low-energy binary selections that satisfy the encoded trade-offs. The same QUBO structure can be adapted to fragment assembly, peptide or protein sequence selection, and multi-objective lead prioritization whenever the decision variables are discrete, and the relevant constraints can be expressed in QUBO or Ising form.

### Practical workflow: From AI scores to QA-driven decisions

To keep this review workflow oriented, QA should be described as a pipeline with explicit interfaces between classical modeling and hardware-constrained optimization. In a typical drug-discovery setting, candidate pools are first generated or scored by classical components such as foundation models, docking, surrogate models for physics-based simulations, or empirical predictors, producing scalar scores, uncertainty estimates, and/or pairwise similarity measures. These heterogeneous outputs then require normalization and objective design so that trade-offs are made explicit (for example, by weighted objectives or by sampling strategies that probe different regions of a Pareto front). Next, practical constraints, such as synthesis or assay budgets, throughput limits, toxicity thresholds, and diversity requirements, must be encoded, usually through penalty terms; importantly, inequality constraints and coupled rules can increase the number of decision variables and therefore amplify embedding overhead.

Once the objective and constraints are defined, the resulting problem graph is embedded onto the QPU topology, and key parameters such as chain strength, anneal schedule/time, number of reads, and gauge choices are selected; this stage is often decisive for both feasibility and performance.^48^ Finally, because sampled solutions can vary across runs and chain breaks can occur, post-processing becomes essential: chain-break resolution, feasibility checks, local refinement, and re-ranking using higher-fidelity classical models are commonly applied, and robustness should be quantified using repeated sampling and aggregation. This end-to-end view clarifies why the value of QA in drug discovery should be judged at the workflow level (decision quality, robustness, and time to target) rather than purely by a single QPU call.

### Problem suitability and boundary conditions for QA

This pipeline also clarifies when QA is likely to be useful and when classical methods remain preferable. QA-suitable problems tend to have discrete binary or categorical decision variables, a meaningful quadratic interaction structure, constraints that can be encoded without excessive auxiliary-variable inflation, and objective functions that can be evaluated cheaply with classical models prior to optimization. Examples include compound subset selection with diversity penalties, modular fragment assembly, combinatorial sequence choice under hard constraints, and resource-limited experiment prioritization. In such settings, the value of QA is not necessarily a guaranteed global optimum but rather the ability to sample diverse, low-energy, feasible solutions that can be post-processed and re-ranked.

By contrast, classical methods are usually preferable for small or weakly constrained instances that exact mixed-integer solvers can solve reliably, continuous optimization tasks such as high-dimensional conformational refinement, objectives that require expensive simulation at each optimization step, or settings in which uncertainty in model outputs dominates the difference between candidate solutions. Classical and quantum-inspired approaches should also be treated as primary baselines whenever the problem formulation is not naturally quadratic or when embedding overhead reduces the QPU’s effective capacity. Thus, the most defensible role for QA in drug discovery is as a selective optimization backend for well-defined, hardware-aware QUBO subproblems rather than as a universal replacement for established classical optimization.

### Evidence from successful QUBO deployments: Transferable lessons from real-world applications

While drug discovery has its own domain constraints, many modeling bottlenecks, multi-objective trade-offs, discrete decisions, inequality constraints, and resource limits are shared across industries. In our published work, we have applied QUBO formulations to multiple real-world, constraint-dominated problems, including electric vehicle (EV) charging station siting (facility location under coverage/cost constraints),^49^ bike rebalancing and last-mile routing (traveling salesman problem [TSP] variants with capacity/operational constraints),^50^^,^^51^ home-care workforce scheduling and emergency patient allocation (resource assignment under demand/feasibility constraints),^52^^,^^53^ and agricultural planning (graph-coloring-like planting decisions and fertilizer allocation under nutrient/budget constraints).^54^^,^^55^

Across these applications, several recurring lessons are directly relevant to drug discovery. Solution quality and feasibility hinge more on constraint engineering, penalty design, scaling, and auxiliary-variable strategy than on the solver choice alone, mirroring drug-discovery portfolio problems where toxicity filters, assay budgets, and diversity requirements must be enforced reliably. Relatedly, many practical tasks cannot be solved well by independent scoring because pairwise interactions matter (e.g., redundancy, compatibility, and mutual exclusivity). This maps cleanly onto compound-set selection, where similarity penalties and coverage/diversity interactions define the objective beyond single-molecule scores.

In routing, scheduling, and allocation settings, larger instances often require decomposition (subproblem solving plus reconciliation) and repeated runs to assess stability, precisely the system-level thinking required when QA is integrated into iterative, AI-driven discovery loops. Finally, “success” in deployed QUBO systems is typically evaluated with operational metrics (feasibility under constraints, robustness to uncertainty, and iteration speed) rather than solver novelty. For drug discovery, the analogous outcomes are improved experimental hit rate under budget, better diversity coverage, and faster iteration through closed-loop cycles. These evidence-based lessons motivate a hardware-aware, workflow-centric evaluation of QA in drug discovery, consistent with the integration and benchmarking considerations discussed in subsequent sections.

### Reporting considerations for a hardware-aware critical review

To meet state-of-the-art expectations in QA studies that are relevant to drug-discovery decision workflows, results should be reported in a way that makes hardware constraints, embedding overhead, and evaluation criteria transparent. At a minimum, authors should specify the optimization backend (QPU generation/topology) and whether a hybrid solver was used, along with any working-graph assumptions that could affect effective capacity. Embedding details should be summarized in terms of the logical-variable count, chain-length characteristics, chain-break handling, and the selection of chain strength, as these factors directly determine the gap between the modeled problem and what is physically realized on hardware. Likewise, the sampling protocol should be described with sufficient detail to assess reproducibility, including the number of reads, anneal schedule/time, gauge strategy (if used), and how repeated runs were aggregated.

Equally important, the objective and constraint design should be made explicit, including how heterogeneous scores were normalized, how penalty weights were selected or calibrated, and whether auxiliary variables were introduced (and what impact they had on the effective problem size). Finally, evaluation should include well-tuned classical baselines and decision-relevant metrics (e.g., solution quality at a fixed time to target, robustness under uncertainty, and sensitivity analyses over penalty and embedding parameters) rather than relying solely on raw energy values. Adopting this reporting practice helps ensure that conclusions about the utility of QA are grounded in workflow-relevant evidence and are comparable across studies. Together, these reporting elements help distinguish genuine workflow-level gains from artifacts of formulation, embedding, or parameter tuning. To translate these reporting considerations into a practical evaluation framework, Table 2 summarizes the key elements that should be specified when benchmarking AI-QA workflows for drug-discovery decision problems, including problem definition, QUBO formulation, solver configuration, embedding overhead, classical baselines, robustness analysis, and decision-relevant performance metrics.

**Table 2:** Proposed benchmarking framework for AI-quantum annealing workflows in drug discovery

Benchmarking element	Recommended reporting items	Purpose for drug-discovery evaluation
Problem definition	specify the decision task, such as compound subset selection, fragment assembly, peptide or protein sequence selection, or multi-objective lead prioritization; report the candidate-pool size and the intended experimental decision	clarifies whether the task is a discrete, constraint-dominated optimization problem suitable for QUBO/Ising formulation
Input data and scores	describe the source of input scores, including AI models, docking, simulation, assay data, or expert rules; report how potency, selectivity, toxicity, developability, uncertainty, and pairwise similarity were estimated or normalized	ensures that optimization performance is interpreted in relation to the quality, uncertainty, and biological relevance of the upstream predictive inputs
QUBO formulation	define binary variables, linear terms, pairwise interaction terms, hard and soft constraints, penalty weights, auxiliary variables, and any quadratization steps	makes the decision model transparent and allows readers to determine whether the encoded objective corresponds to a realistic drug-discovery decision
Solver and hardware configuration	report the optimization backend, such as QPU, hybrid solver, digital annealer, quantum-inspired solver, or classical solver; for QA hardware, specify processor generation, topology, anneal schedule or time, number of reads, gauge strategy, and post-processing	distinguishes solver-level effects from formulation effects and supports reproducible comparison across hardware and software backends
Embedding and scalability	for hardware QA, report logical-variable count, physical-qubit count, chain-length distribution, chain strength, chain-break frequency, and embedding success rate; for hybrid solvers, describe decomposition strategy	quantifies the gap between the intended QUBO model and the physically or algorithmically realized optimization problem.
Classical and quantum-inspired baselines	compare against well-tuned baselines, such as greedy ranking, diversity-filtered selection, genetic algorithms, simulated annealing, Bayesian optimization, mixed-integer programming when applicable, and digital or quantum-inspired annealers	prevents overinterpretation of annealing results and identifies whether QA provides workflow-level value beyond established optimization methods
Evaluation metrics	report objective value, feasibility rate, constraint violation rate, diversity or coverage, enrichment of high-quality candidates, synthesis or assay cost, time to target, and downstream hit rate when available	aligns computational performance with practical discovery outcomes rather than relying solely on raw energy values
Robustness and sensitivity	perform repeated runs and report variability across random seeds, gauges, embeddings, penalty weights, chain strengths, and uncertainty perturbations in input scores	assesses whether selected candidates are stable enough to guide costly synthesis, assay, or in vivo decisions
Workflow integration	describe how optimized solutions are post-processed, re-ranked, experimentally validated, and reintegrated into active-learning or closed-loop design cycles	evaluates annealing as part of an end-to-end discovery workflow rather than as an isolated solver demonstration

AI, artificial intelligence; QA, quantum annealing; QPU, quantum processing unit; QUBO, quadratic unconstrained binary optimization.

This framework summarizes the minimum information that should be reported when evaluating annealing-based optimization as a decision layer within AI-enabled drug-discovery workflows.

## Applications of QA in drug discovery

The practical relevance of QA to drug discovery lies not in abstract demonstrations of quantum behavior but in its ability to address specific optimization tasks that arise downstream of AI-enabled candidate generation. These tasks are typically discrete, constraint dominated, and combinatorial in nature, and they often determine experimental efficiency and development trajectory. In this section, we review application areas in which QA has been explored or proposed as an optimization layer, emphasizing realistic use cases, formulation strategies, and integration with existing computational workflows.

### Compound selection and library pruning

One of the most immediate applications of QA in drug discovery is compound selection. Modern AI workflows can generate or score tens of thousands to millions of candidate molecules, but experimental resources typically allow only a small fraction to be synthesized or tested. Selecting subsets that balance predicted activity, chemical diversity, risk, and cost is a canonical combinatorial optimization problem.

**Figure 1:**
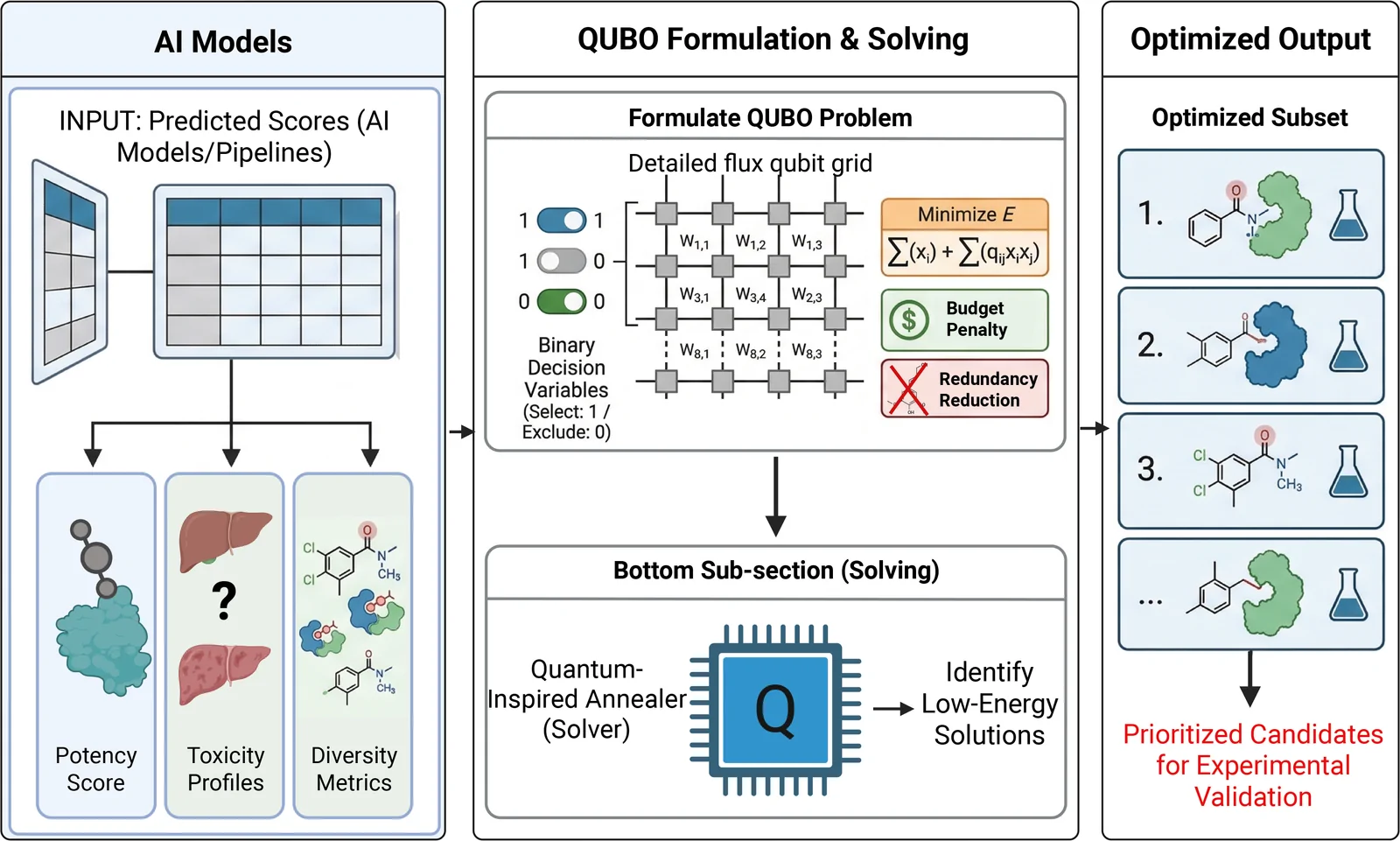
Compound subset selection using QUBO-based optimization Predicted scores generated by AI models or computational pipelines (e.g., potency, toxicity, and diversity metrics) are used as inputs to formulate a QUBO problem. Binary decision variables represent whether individual compounds are selected or excluded, while penalty terms encode practical constraints such as experimental budget and redundancy reduction. Quantum annealing or quantum-inspired annealing is then applied to identify low-energy solutions corresponding to optimized subsets for synthesis and experimental testing.

In this setting, binary variables represent whether individual compounds are selected, while objective terms encode predicted properties such as potency, selectivity, or novelty derived from AI models.^56^ Pairwise interaction terms can penalize redundancy by discouraging the simultaneous selection of highly similar compounds, while constraints enforce limits on budget, synthesis capacity, or assay throughput. This formulation naturally maps to a QUBO, enabling QA to sample low-energy solutions across a large combinatorial subspace (Figure 1).

**Figure 2:**
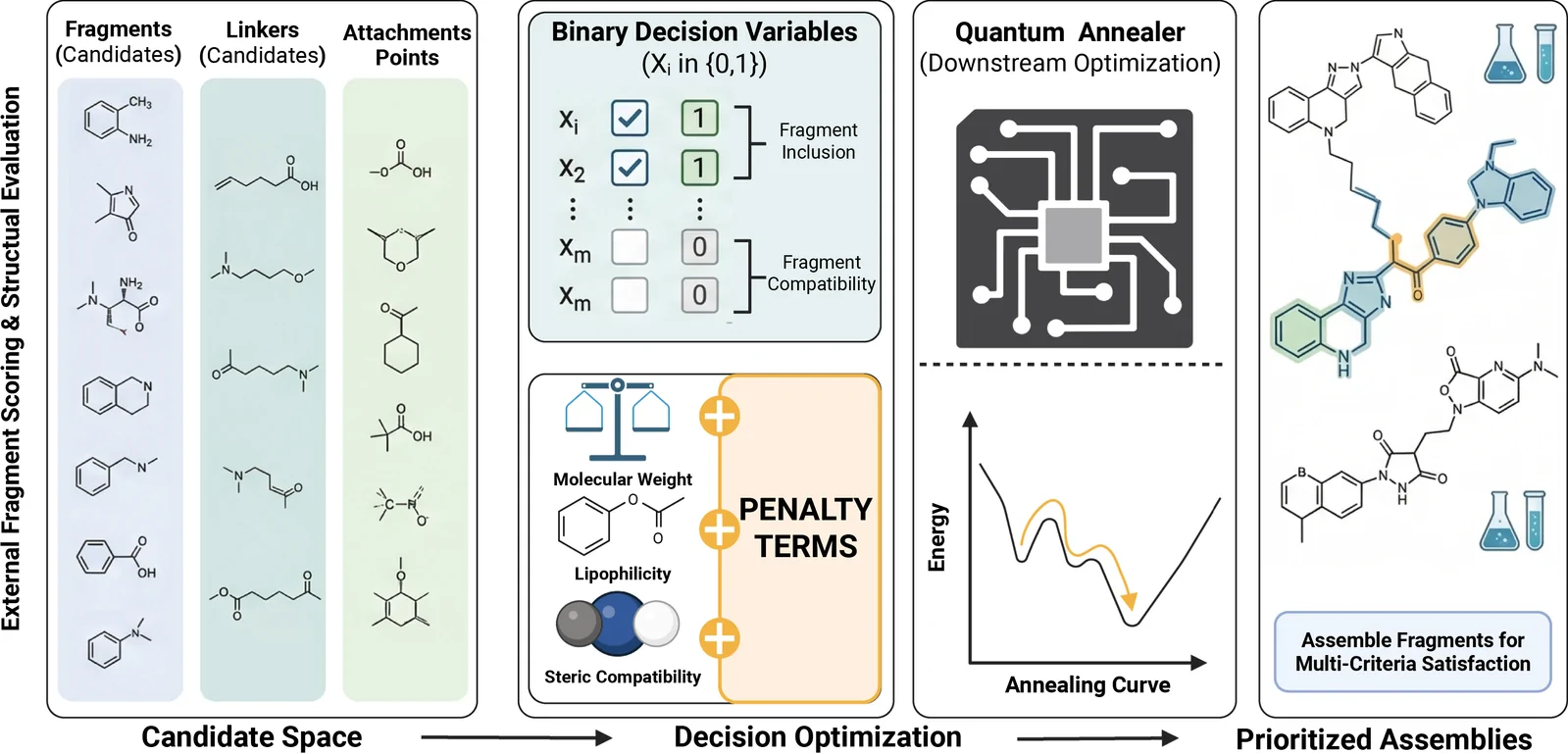
Fragment-based drug design as a combinatorial optimization problem This figure illustrates that candidate molecular fragments, linkers, and attachment points define a large combinatorial design space. Fragment inclusion and compatibility are represented as binary decision variables, while physicochemical and chemical feasibility constraints (e.g., molecular weight limits, lipophilicity, and steric compatibility) are incorporated as penalty terms within a QUBO formulation. Annealing-based optimization identifies fragment assemblies that simultaneously satisfy multiple design criteria. Fragment scoring and structural evaluation are performed externally, with annealing applied only to the downstream decision optimization.

Compared with ranking-based approaches, subset optimization explicitly accounts for interactions among selected candidates rather than treating each compound independently.^57^ This distinction is particularly important when diversity or coverage of chemical space is a priority. While classical heuristics can address similar problems, performance often degrades as the number of candidates and constraints increases, motivating the investigation of annealing-based approaches for early hit triage and follow-up screening.

### Fragment-based design and combinatorial assembly

Beyond subset selection, fragment-based design introduces modular assembly decisions that further amplify combinatorial complexity. Fragment-based drug discovery introduces additional layers of combinatorial complexity. Decisions regarding which fragments to combine, which linkers to use, and which attachment points are compatible give rise to large, discrete search spaces. AI models can score fragment-target interactions or propose candidate assemblies, but selecting optimal combinations under chemical and geometric constraints remains challenging.

QA formulations for fragment-based design typically encode fragment inclusion as binary variables, with pairwise terms representing compatibility, steric feasibility, or shared chemical liabilities.^58^ Constraints can enforce limits on molecular weight, lipophilicity, polar surface area, or synthetic accessibility. In this context, annealing-based optimization can assist with identifying feasible and high-scoring assemblies that satisfy multiple design criteria simultaneously (Figure 2).

Although these approaches remain exploratory, they illustrate how QA can be applied to modular design problems in which discrete choices dominate. Importantly, QA does not replace fragment scoring, docking, or structural evaluation but instead operates on outputs generated by those methods to optimize combinatorial decisions.

### Peptide and protein design optimization

**Figure 3:**
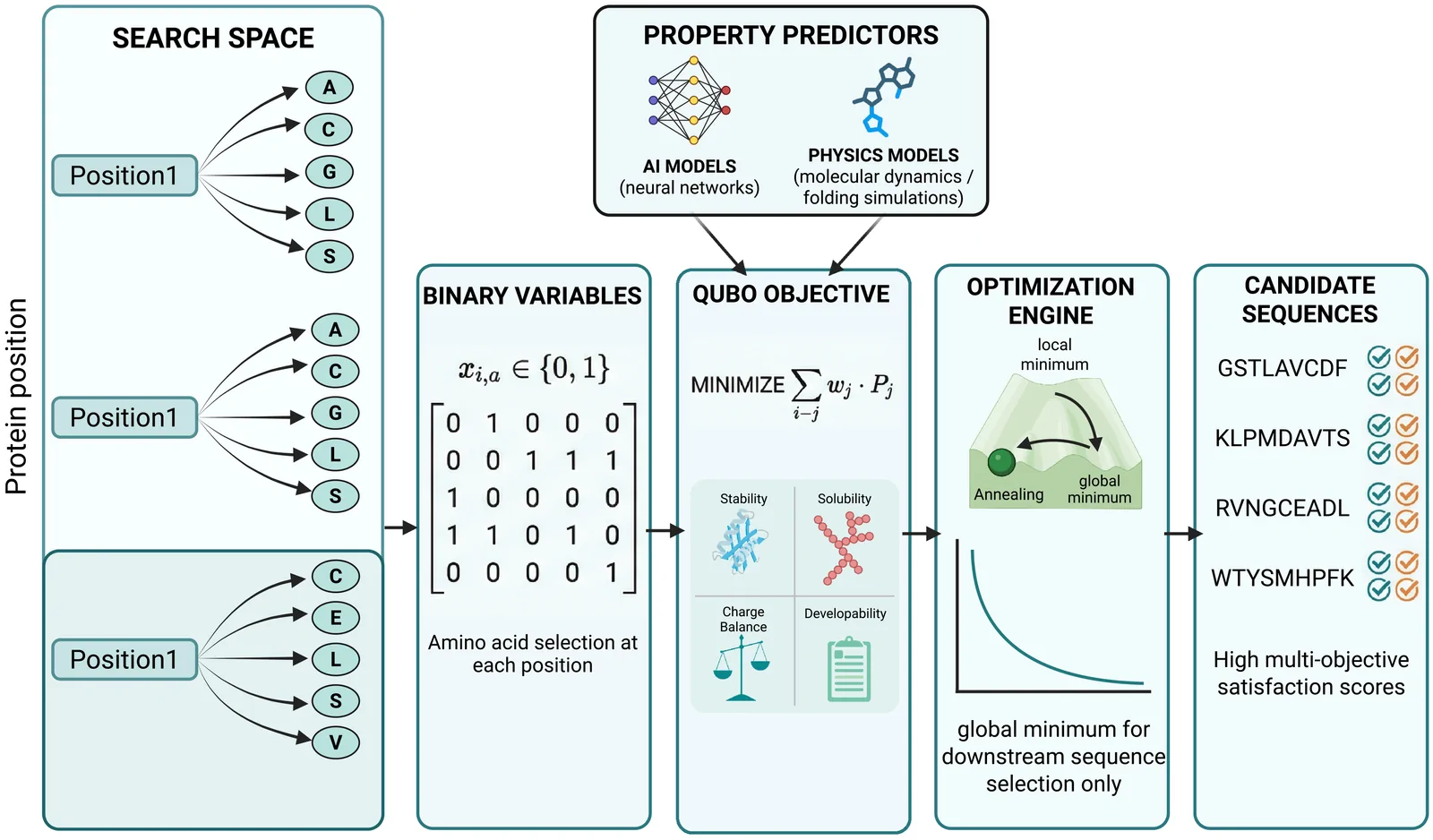
Constrained peptide and protein sequence optimization At each sequence position, multiple amino acid choices define a discrete combinatorial search space. Binary variables encode sequence selection decisions, while constraints related to stability, solubility, charge balance, and developability are incorporated into a QUBO objective. Predicted properties are provided by AI or physics-based models, and annealing is used solely to optimize sequence selection under these constraints, yielding candidate sequences that satisfy multiple design objectives simultaneously.

Peptide and protein design present particularly compelling use cases for QA due to the discrete nature of sequence decisions. Side-chain selection, residue substitution, and motif inclusion can be naturally represented as binary or categorical variables. Even when restricting attention to a subset of residues, the resulting combinatorial space can be enormous, rendering exhaustive search impractical (Figure 3).

In protein design workflows, AI models increasingly provide estimates of stability, binding affinity, or functional likelihood for candidate sequences. QA can then be used to select residue combinations that optimize these scores while satisfying structural or functional constraints. Side-chain packing, in particular, has long been recognized as a nondeterministic polynomial time (NP)-hard problem, and annealing-based approaches have been explored as alternatives or complements to classical heuristics.^59^

Similarly, peptide optimization tasks, such as balancing binding affinity against proteolytic stability, solubility, or immunogenicity, can be framed as multi-objective optimization problems with discrete variables. In these contexts, QA may assist with identifying Pareto-optimal or near-optimal solutions that would be difficult to obtain using greedy or local search methods alone.^11^

### Multi-objective lead optimization under constraints

Lead optimization represents one of the most challenging stages of drug discovery, as improvements in potency often come at the cost of pharmacokinetics, safety, or developability. AI models can predict individual properties with increasing sophistication, but integrating these predictions into actionable decisions remains non-trivial. Multi-objective optimization typically involves trade-offs that are difficult to reduce to a single score.

QA can be applied to this problem by encoding multiple objectives into a composite energy function, with adjustable weights reflecting relative priorities.^11^ Hard constraints, such as toxicity thresholds, formulation requirements, or chemical liabilities, can be enforced through penalty terms, while soft objectives contribute to the overall score. By sampling from the energy landscape, QA can identify candidate sets that balance competing objectives rather than optimizing a single metric. This approach is particularly relevant when optimization decisions must be made under strict resource constraints, such as selecting a limited number of leads for costly *in vivo* or investigational new drug (IND)-enabling studies.

### Case studies of annealing-based optimization in drug discovery

Several proof-of-concept studies demonstrate that annealing-based optimization can be applied to discrete decision problems in drug discovery. Rather than demonstrating end-to-end automation, these examples highlight specific optimization tasks in which QUBO formulations provide a natural abstraction for integrating model outputs and practical constraints.

#### Case 1: Compound subset selection under experimental constraints

In one representative scenario, predicted activity and toxicity scores generated by classical machine learning models are combined with budgetary and diversity constraints to formulate a compound selection problem as a QUBO. Binary decision variables indicate whether individual compounds are selected for synthesis or testing, while penalty terms constrain experimental throughput and redundancy. Annealing-based solvers are then used to identify low-energy subsets that balance predicted performance with practical feasibility, demonstrating improved prioritization relative to heuristic or greedy selection strategies.^59^

#### Case 2: Fragment and combinatorial assembly optimization

A second class of examples focuses on fragment-based design, where candidate fragments and linkers define a large combinatorial design space. Here, compatibility constraints, physicochemical limits, and scoring functions derived from docking or learned models are encoded within a QUBO formulation. Annealing-based optimization enables efficient exploration of feasible fragment assemblies under multiple constraints, producing candidate designs that satisfy joint criteria without exhaustively enumerating all combinations.^60^

#### Case 3: Constrained sequence selection for peptides and proteins

Annealing-based methods have also been explored for discrete sequence optimization problems, such as peptide or protein variant selection. At each sequence position, binary variables represent allowable amino acid choices, while constraints related to stability, solubility, or developability are incorporated as penalties. Predicted properties are provided externally by AI or physics-based models, and annealing is applied solely to optimize sequence selection under these constraints, yielding candidate sequences that satisfy multiple design objectives simultaneously.^61^

**Figure 4:**
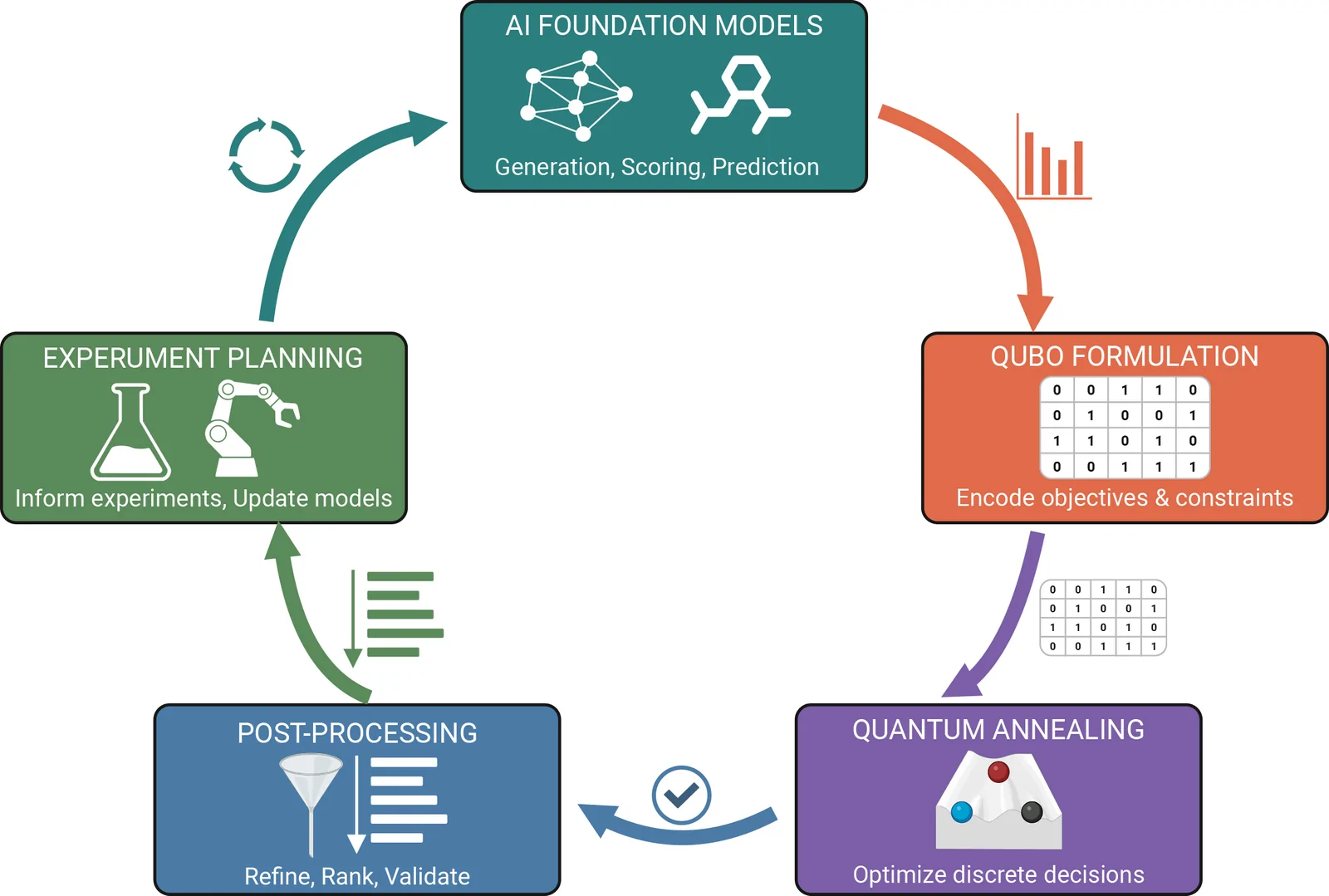
Hybrid AI-quantum workflow in drug discovery A common architectural pattern for integrating quantum annealing into AI-driven drug discovery follows an AI → optimization → AI loop, as summarized in this figure.

Together, these mini-case studies demonstrate that annealing-based optimization is most naturally applied as a downstream decision-making tool, operating on discrete choices derived from predictive models. The common architectural patterns emerging from these applications are abstracted in Figure 4.

### Evaluation, benchmarking, and current limitations

Evaluating the performance of QA in drug discovery remains challenging. Results are highly sensitive to problem formulation, penalty weight selection, and embedding choices, making direct comparisons with classical methods difficult.^62^ Moreover, many published studies focus on simplified benchmarks that may not reflect the complexity of real-world drug-discovery tasks.

Recent benchmarking studies reinforce this need for problem-specific evaluation. Comparative analyses of D-Wave hybrid solvers against classical solvers such as CPLEX, Gurobi, and IPOPT show that hybrid annealing can be competitive for some binary or quadratic problem classes but that performance deteriorates or becomes inconclusive for more general linear, mixed-integer, or heavily constrained formulations.^63^ Conversely, other recent dense-QUBO benchmarking studies report favorable accuracy and time to solution for state-of-the-art annealing-based workflows under selected conditions.^64^ Taken together, these results suggest that the central question is not whether QA is categorically better or worse than classical optimization but which mathematical structures, constraint profiles, and workflow objectives make annealing-based optimization practically worthwhile.

Reproducibility and robustness are additional concerns. Annealing outcomes can vary across runs, necessitating repeated sampling and statistical aggregation. From a practical standpoint, the value of QA should therefore be assessed not in isolation but in terms of its contribution to overall workflow efficiency and decision quality.^17^^,^^18^^,^^25^^,^^65^^,^^66^

Taken together, current applications of QA in drug discovery should be viewed as exploratory but instructive. They demonstrate how annealing-based optimization can be mapped to concrete decision problems and integrated with AI-driven pipelines. The key question is not whether QA universally outperforms classical heuristics but whether it offers advantages for specific, well-defined optimization tasks. The next section examines how these application-level insights inform the design of hybrid AI-quantum workflows and system-level considerations.

### Hybrid AI-quantum workflows and system-level considerations

Building on the application examples discussed above, this section examines how annealing-based optimization can be systematically integrated into AI-driven drug-discovery pipelines. Rather than focusing on individual algorithms or solvers, we emphasize recurring architectural patterns, interface boundaries, and design considerations that enable hybrid workflows across diverse application contexts. These patterns provide a practical framework for translating problem formulations into deployable, end-to-end discovery systems.

As for the architectural patterns for hybrid workflows, in this modular design (Figure 4), AI foundation models are first used to generate, score, or evaluate large candidate pools based on molecular, protein, or multimodal representations. These outputs, typically scalar property predictions, uncertainty estimates, or pairwise similarity measures, are then translated into a QUBO formulation that encodes downstream decision objectives and practical constraints. QA is applied selectively at this stage to optimize discrete decision variables, such as compound subset selection or combinatorial configuration choices. The resulting optimized decisions are subsequently post-processed and reintegrated into downstream experimental planning or subsequent AI-driven design cycles, forming a closed-loop hybrid workflow.^65^

This modular architecture preserves separation of concerns. AI models remain responsible for learning from data and making predictions, while QA focuses exclusively on decision optimization. Such separation simplifies validation and reduces the risk of conflating predictive performance with optimization efficacy. Importantly, it also allows components to be upgraded independently as models, hardware, or algorithms improve.

### Problem decomposition and selective quantum use

Effective hybrid workflows require careful problem decomposition. Not all steps in drug discovery benefit from QA, and indiscriminate use can introduce unnecessary complexity. Instead, QA should be applied selectively to subproblems where (1) decision variables are discrete, (2) constraints dominate the feasible solution space, and (3) objective functions can be evaluated efficiently using classical computation.^67^

Examples include subset selection under budget constraints, combinatorial assembly of modular designs, and multi-objective lead prioritization with hard exclusion criteria. By contrast, tasks involving continuous optimization, expensive simulations, or high uncertainty may be better addressed using classical methods alone. Identifying appropriate cut points between classical and quantum components is therefore a central system-level design decision.

### When QA is useful—and when it is not

QA is most likely to be useful when the optimization problem can be expressed as a discrete decision task with explicit constraints and meaningful pairwise interactions. In drug discovery, such settings include compound subset selection under synthesis or assay-budget constraints, fragment assembly with compatibility rules, peptide or protein sequence selection with developability requirements, and lead-prioritization problems that require balancing potency, safety, diversity, and resource allocation. In these cases, the value of QA does not arise from predicting molecular properties but from exploring combinations of binary decisions after candidate scores, uncertainty estimates, and constraints have already been generated by AI models, docking, simulation, or experimental assays.

Several problem characteristics make a task more suitable for QA or quantum-inspired annealing. First, the decision variables should be naturally binary or categorical, such as selecting or excluding a compound, choosing a fragment, or assigning an amino acid option at a sequence position. Second, the feasible solution space should be strongly shaped by constraints, including experimental throughput, synthesis cost, toxicity thresholds, chemical diversity requirements, mutual exclusivity, or portfolio balance. Third, pairwise interactions should matter; for example, two individually promising compounds may be redundant because of high chemical similarity, or two fragments may be incompatible despite favorable individual scores. Fourth, the objective function should be inexpensive enough to evaluate repeatedly once the input scores and constraint terms have been defined. Finally, the candidate pool should usually be prefiltered to a tractable size before QUBO construction, as embedding overhead and hardware limitations can make direct treatment of very large dense problems impractical.

By contrast, QA is unlikely to be the preferred approach when the dominant challenge is continuous optimization, high-fidelity molecular simulation, uncertain property prediction, or mechanistic modeling. Tasks such as molecular dynamics, binding free energy estimation, protein structure prediction, reaction pathway simulation, or pharmacokinetic model fitting generally require continuous variables, physics-based computation, or statistical inference rather than binary decision optimization. Similarly, when candidate scores are highly uncertain or poorly calibrated, improving the predictive model may be more important than optimizing downstream selections. QA may also offer limited practical value when a problem is small, weakly constrained, or well solved by simple greedy ranking, clustering, mixed-integer programming, or other classical heuristics.

A further limitation arises when the QUBO graph is too dense or requires many auxiliary variables to encode constraints. In such cases, minor embedding may substantially increase the number of physical qubits required, introduce long chains, increase chain-break frequency, and reduce reproducibility. For near-term hardware, these considerations often favor decomposition, hybrid solvers, quantum-inspired digital annealers, or well-tuned classical methods. Therefore, the relevant question is not whether QA is universally advantageous but whether a given drug-discovery decision problem has the structure, scale, and constraint profile for which annealing-based optimization can provide incremental workflow-level benefit.

A practical decision rule is to use QA selectively when the problem is discrete, constraint rich, interaction dependent, and embedded within a broader AI-enabled workflow in which predictive modeling and experimental validation remain classical or empirical. Under this framing, QA functions as a specialized decision-optimization layer, not as a general-purpose replacement for AI models, physics-based simulation, or medicinal chemistry judgment.

### Preprocessing, embedding, and post-processing

Translating AI outputs into QUBO formulations requires non-trivial preprocessing. Scores from heterogeneous models must be normalized, constraints must be encoded as penalties with appropriate weights, and problem graphs must be embedded onto the connectivity, supported by available annealing hardware. These steps can significantly affect optimization outcomes and are often more consequential than the annealing process itself.

Post-processing is equally important. Because annealing outcomes can vary across runs, hybrid workflows typically involve repeated sampling and aggregation to identify robust solutions. Classical post-processing may include local refinement, feasibility checks, or re-ranking using higher-fidelity models. From a system perspective, these steps help mitigate noise and improve reproducibility, but they also add latency and computational overhead that must be accounted for in workflow design.

### Integration with experimental and automated platforms

Hybrid AI-quantum workflows are most impactful when integrated with experimental feedback loops, including automated synthesis, high-throughput screening, and active learning systems. In such closed-loop designs, optimization outputs inform experimental decisions, and experimental results are used to update AI models and objective functions iteratively.^68^ QA may thus contribute indirectly to learning efficiency by improving the quality of decisions made at each iteration.

From a system engineering standpoint, this integration raises additional considerations related to data flow, timing, and decision authority. Optimization outputs must be interpretable and actionable by experimental platforms, and failure modes must be detectable to prevent propagation of erroneous decisions. These requirements favor conservative, transparent optimization formulations over aggressive or opaque strategies.

### Classical and quantum-inspired baselines for fair comparison

A rigorous assessment of QA in drug-discovery workflows requires comparison against well-tuned classical and quantum-inspired baselines rather than against naive ranking procedures alone. Because many drug-discovery optimization tasks can be formulated as discrete or mixed discrete-continuous decision problems, several established optimization strategies may provide strong performance depending on the problem structure, objective-function cost, and constraint complexity. QA should therefore be evaluated as one optimization backend within a broader solver ecosystem rather than as an isolated or intrinsically superior computational paradigm.

For compound subset selection, a minimal baseline is greedy ranking based on predicted utility scores, optionally followed by diversity filtering or clustering-based selection. Such methods are simple, interpretable, and often effective when candidate scores dominate the decision process and pairwise interactions are weak. However, greedy approaches may become suboptimal when selected candidates interact through redundancy, mutual exclusivity, budget limits, or portfolio-balance constraints. In these cases, stronger classical baselines should be included, such as genetic algorithms, simulated annealing, tabu search, mixed-integer programming, or other combinatorial optimization methods that can explicitly search across constrained solution spaces.

Bayesian optimization and reinforcement learning provide additional baselines in settings where objective functions are expensive, uncertain, or sequentially updated. Bayesian optimization is particularly relevant when each evaluation requires costly simulation or experimental feedback, whereas reinforcement learning may be useful when candidate generation and optimization are coupled through sequential design policies. However, both approaches often require scalarized reward or acquisition functions, and their performance can be sensitive to the choice of objective weighting, uncertainty model, and constraint-handling strategy. These limitations should be considered when comparing them with QUBO-based optimization.

Quantum-inspired methods, including digital annealers and specialized classical hardware for QUBO, are especially important comparators because they share the same or similar mathematical interface as QA while avoiding the constraints of quantum hardware, such as minor embedding, chain breaks, analog control errors, and limited effective problem size. In near-term applications, these methods may provide greater scalability or reproducibility for certain problem classes and may serve either as practical substitutes or as intermediate benchmarks before deployment on QA hardware.

Fair comparison should therefore be based on workflow-relevant metrics rather than raw energy values alone. Relevant criteria include feasibility rate, best and average objective value, diversity or coverage of the selected portfolio, robustness across repeated runs, sensitivity to penalty weights, computational cost, time to target, and downstream experimental relevance, such as predicted hit enrichment or reduction in synthesis burden. When QA is evaluated, the comparison should also report the embedding overhead, logical and physical variable counts, chain-break frequency, post-processing procedures, and whether hybrid solvers were used. Such reporting allows readers to distinguish genuine workflow-level benefit from artifacts of formulation, parameter tuning, or hardware-specific implementation.

Overall, the goal is not to demonstrate universal quantum advantage, which has not been established for drug-discovery-relevant optimization problems, but to identify problem regimes in which annealing-based optimization may provide incremental value over strong classical and quantum-inspired alternatives. These regimes are most likely to involve discrete decision variables, dense pairwise interactions, explicit resource constraints, and the need to balance multiple competing objectives within a closed-loop AI-enabled discovery workflow.

### Quantum-inspired and classical alternatives

It is important to situate QA within a broader ecosystem of optimization technologies. Quantum-inspired classical methods, including digital annealers and specialized hardware accelerators, implement similar energy-minimization strategies without relying on quantum coherence.^69^ These approaches often offer greater scalability and stability in the near term and can serve as benchmarks or substitutes when quantum hardware is unavailable or impractical.

From a systems-level perspective, hybrid workflows should therefore be designed to be hardware agnostic, allowing optimization backends to be swapped or combined as appropriate. This flexibility reduces lock-in risk and supports incremental adoption, enabling organizations to evaluate QA pragmatically alongside classical alternatives.^70^

### Evaluation metrics and operational considerations

Assessing the value of hybrid AI-quantum workflows requires metrics that go beyond raw optimization performance. Relevant criteria include decision quality, robustness to uncertainty, experimental efficiency, and overall impact on project timelines. Because QA is typically applied to subproblems rather than entire pipelines, its contribution should be evaluated in terms of marginal gains relative to baseline workflows.^62^

Operational considerations such as cost, accessibility, and integration effort are also critical. Current QA platforms are typically accessed via cloud services, introducing latency and data governance considerations. These factors further reinforce the need for selective, well-justified use of quantum components within hybrid systems.

In summary, hybrid AI-quantum workflows offer a pragmatic pathway for exploring the potential of QA in drug discovery. Their success depends less on the performance of quantum hardware than on thoughtful systems-level design, problem decomposition, and integration with existing computational and experimental infrastructures. The final section of this review examines broader limitations, benchmarking challenges, and future outlooks that shape the long-term role of hybrid optimization strategies in drug discovery.

## Limitations, benchmarking, and outlook

Despite growing interest in hybrid AI-quantum workflows, substantial constraints currently limit the practical impact of QA in drug discovery. These constraints extend beyond hardware performance to include problem formulation, benchmarking rigor, and workflow integration. Transparent evaluation across these dimensions is essential to prevent overinterpretation of early or problem-specific successes.

Hardware scale and connectivity remain fundamental limitations. After accounting for embedding overhead, current annealers support a restricted number of effective binary variables, constraining the size and structural fidelity of real-world optimization problems. Sparse connectivity necessitates non-trivial embedding strategies that may distort problem structure and amplify noise, often requiring aggressive decomposition or simplification of drug-discovery tasks before mapping them to annealing hardware.

Stochastic variability and reproducibility present additional challenges. Annealing outcomes can differ across runs due to hardware noise and probabilistic dynamics, necessitating repeated sampling, aggregation, and classical post-processing.^62^ While stochastic optimization is not inherently problematic, the high downstream cost of drug-discovery decisions demands robustness, interpretability, and conservative validation relative to deterministic classical baselines.

A further central issue is the absence of demonstrated, problem-agnostic quantum advantage for drug-discovery-relevant tasks. Reported performance improvements are highly formulation dependent and sensitive to parameter tuning, embedding strategy, and implementation maturity.^25^ Meaningful claims therefore require benchmarking against well-optimized classical heuristics under comparable evaluation criteria.^21^ Without standardized baselines and reproducible benchmarking protocols, comparative assessments remain difficult to interpret.

This mixed evidence is important for drug discovery because many relevant formulations contain both favorable QUBO-like structure (binary choices and pairwise interactions) and unfavorable features (heterogeneous constraints, noisy predictive scores, and auxiliary variables). A critical review should therefore report not only successful demonstrations but also negative or inconclusive results, as these clarify the boundaries of current hardware and hybrid solvers and prevent overgeneralization from highly tailored benchmarks.^63^^,^^64^

Benchmark design itself represents a methodological bottleneck. Many existing studies rely on stylized or small-scale problems that do not capture the multi-objective trade-offs, noisy predictions, and heterogeneous constraints characteristic of real discovery workflows.^17^^,^^71^ Robust evaluation should incorporate realistic constraints, uncertainty-aware objectives, and decision-relevant metrics such as experimental efficiency, portfolio diversity, or downstream attrition risk.

From a systems perspective, integration costs and operational complexity are non-trivial. Hybrid AI-quantum pipelines require expertise spanning machine learning, combinatorial optimization, hardware interfacing, and domain-specific constraint modeling. Data governance considerations, cloud latency, and regulatory oversight further influence practical deployment. As a result, early adoption is likely to remain selective and problem driven rather than industry wide. For example, recent biomedical applications have used QA-based QUBO formulations for feature selection in single-cell RNA sequencing data, illustrating how annealing-based optimization may support high-dimensional biological data analysis beyond purely chemical design tasks.^72^

Looking ahead, near- to mid-term progress will likely emerge from incremental, architecture-level advances rather than hardware breakthroughs alone. Improvements in embedding strategies, hybrid classical-quantum decomposition, and modular workflow design may gradually expand the class of tractable subproblems.^65^ Equally important will be the development of hardware-agnostic interfaces and standardized evaluation frameworks that enable objective comparison across optimization paradigms.

Ultimately, the long-term relevance of QA must be assessed relative to advances in classical and quantum-inspired optimization methods. Many capabilities attributed to annealing—parallel exploration of rugged energy landscapes and explicit encoding of discrete constraints—are also being pursued through specialized classical hardware and algorithms.^66^^,^^69^ Maintaining a hardware-agnostic, outcome-oriented perspective will therefore be critical.

In the near term, QA is unlikely to function as a transformative standalone technology in drug discovery. Its most realistic role is as a complementary optimization layer for carefully defined combinatorial decision problems within AI-enabled workflows. Real progress will depend less on claims of quantum superiority than on rigorous benchmarking, disciplined system integration, and clear evidence of incremental value within real-world discovery pipelines.

## Acknowledgments

The authors acknowledge financial support from the 10.13039/100020595National Science and Technology Council, Taiwan (NSTC; grant no. NSTC 114-2321-B-195-002 and NSTC 114-2314-B-195-012-MY3).

## Declaration of interests

The authors declare no competing interests.

## Declaration of generative AI and AI-assisted technologies in the writing process

A first draft of the figures was created with the help of BioRender with publication licensure agreements. The figures were subsequently redrawn using conventional graphical tools. The authors reviewed and edited the output as needed and take full responsibility for the content of the published article.

## References

[1] Vamathevan J., Clark D., Czodrowski P., Dunham I., Ferran E., Lee G., Li B., Madabhushi A., Shah P., Spitzer M., Zhao S. (2019). Applications of machine learning in drug discovery and development. Nat. Rev. Drug Discov..

[2] Walters W.P., Murcko M. (2020). Assessing the impact of generative AI on medicinal chemistry. Nat. Biotechnol..

[3] Mak K.-K., Pichika M.R. (2019). Artificial intelligence in drug development: Present status and future prospects. Drug Discov. Today.

[4] Schneider G. (2018). Automating drug discovery. Nat. Rev. Drug Discov..

[5] Chen H., Engkvist O., Wang Y., Olivecrona M., Blaschke T. (2018). The rise of deep learning in drug discovery. Drug Discov. Today.

[6] Walters W.P., Barzilay R. (2021). Applications of deep learning in molecule generation and molecular property prediction. Acc. Chem. Res..

[7] Rives A., Meier J., Sercu T., Goyal S., Lin Z., Liu J., Guo D., Ott M., Zitnick C.L., Ma J., Fergus R. (2021). Biological structure and function emerge from scaling unsupervised learning to 250 million protein sequences. Proc. Natl. Acad. Sci. USA.

[8] Jumper J., Evans R., Pritzel A., Green T., Figurnov M., Ronneberger O., Tunyasuvunakool K., Bates R., Žídek A., Potapenko A. (2021). Highly accurate protein structure prediction with AlphaFold. Nature.

[9] Tong X., Liu X., Tan X., Li X., Jiang J., Xiong Z., Xu T., Jiang H., Qiao N., Zheng M. (2021). Generative models for de novo drug design. J. Med. Chem..

[10] Brown N., Fiscato M., Segler M.H.S., Vaucher A.C. (2019). GuacaMol: Benchmarking models for de novo molecular design. J. Chem. Inf. Model..

[11] Lusher S.J., McGuire R., Azevedo R., Boiten J.-W., van Schaik R.C., de Vlieg J. (2011). A molecular informatics view on best practice in multi-parameter compound optimization. Drug Discov. Today.

[12] Holland J.H. (1992).

[13] Bertsimas D., Tsitsiklis J. (1993). Simulated annealing. Stat. Sci..

[14] Shahriari B., Swersky K., Wang Z., Adams R.P., de Freitas N. (2016). Taking the human out of the loop: A review of Bayesian optimization. Proc. IEEE.

[15] Snoek J., Larochelle H., Adams R.P. (2012). Practical Bayesian optimization of machine learning algorithms. Adv. Neural Inf. Process. Syst..

[16] Frazier P.I. (2018). A tutorial on Bayesian optimization. arXiv.

[17] Hauke P., Katzgraber H.G., Lechner W., Nishimori H., Oliver W.D. (2020). Perspectives of quantum annealing: Methods and implementations. Rep. Prog. Phys..

[18] Lucas A. (2014). Ising formulations of many NP problems. Front. Phys..

[19] Kadowaki T., Nishimori H. (1998). Quantum annealing in the transverse Ising model. Phys. Rev. E.

[20] Santoro G.E., Tosatti E. (2006). Optimization using quantum mechanics: Quantum annealing through adiabatic evolution. J. Phys. A.

[21] Preskill J. (2018). Quantum computing in the NISQ era and beyond. Quantum.

[22] Ur Rasool R., Ahmad H.F., Rafique W., Qayyum A., Qadir J., Anwar Z. (2023). Quantum computing for healthcare: A review. Future Internet.

[23] Ciacco A., Guerriero F., Macrina G. (2025). Review of quantum algorithms for medicine, finance and logistics. Soft Comput..

[24] Bukkarayasamudram V.K., Reddy P.C.S., Arun Kumar K., Jagadish R.M., Sharma S., Prasad M.L., Sucharitha Y., Tayubi I.A., Thakur G.K. (2025). Quantum computing revolution in healthcare: A systematic review of applications, issues and future directions. Artif. Intell. Rev..

[25] McGeoch C.C. (2020). Theory versus practice in annealing-based quantum computing. Theor. Comput. Sci..

[26] Gaulton A., Hersey A., Nowotka M., Bento A.P., Chambers J., Mendez D., Mutowo P., Atkinson F., Bellis L.J., Cibrián-Uhalte E. (2017). The ChEMBL database in 2017. Nucleic Acids Res..

[27] Yang K.K., Wu Z., Bedbrook C.N., Arnold F.H. (2018). Learned protein embeddings for machine learning. Bioinformatics.

[28] Tunyasuvunakool K., Adler J., Wu Z., Green T., Zielinski M., Žídek A., Bridgland A., Cowie A., Meyer C., Laydon A. (2021). Highly accurate protein structure prediction for the human proteome. Nature.

[29] Elnaggar A., Heinzinger M., Dallago C., Rehawi G., Wang Y., Jones L., Gibbs T., Feher T., Angerer C., Steinegger M. (2022). ProtTrans: Towards cracking the language of life's code through self-supervised deep learning and high performance computing. IEEE Trans. Pattern Anal. Mach. Intell..

[30] Torrisi M., Pollastri G., Le Q. (2020). Deep learning methods in protein structure prediction. Comput. Struct. Biotechnol. J..

[31] Gilmer J., Schoenholz S.S., Riley P.F., Vinyals O., Dahl G.E. (2017). Proceedings of the 34th International Conference on Machine Learning (ICML).

[32] Ho J., Jain A., Abbeel P. (2020). Denoising diffusion probabilistic models. Adv. Neural Inf. Process. Syst..

[33] Jin W., Barzilay R., Jaakkola T. (2018). Proceedings of the 35th International Conference on Machine Learning (ICML).

[34] Wang Y., Guo M., Chen X., Ai D. (2025). Screening of multi deep learning-based de novo molecular generation models and their application for specific target molecular generation. Sci. Rep..

[35] Zhavoronkov A., Ivanenkov Y.A., Aliper A., Veselov M.S., Aladinskiy V.A., Aladinskaya A.V., Terentiev V.A., Polykovskiy D.A., Kuznetsov M.D., Asadulaev A. (2019). Deep learning enables rapid identification of potent DDR1 kinase inhibitors. Nat. Biotechnol..

[36] Olivecrona M., Blaschke T., Engkvist O., Chen H. (2017). Molecular de-novo design through deep reinforcement learning. J. Cheminform..

[37] Zitnik M., Nguyen F., Wang B., Leskovec J., Goldenberg A., Hoffman M.M. (2019). Machine learning for integrating data in biology and medicine: Principles, practice, and opportunities. Inf. Fusion.

[38] Garau-Luis J.J., Bordes P., Gonzalez L., Roller M., de Almeida B., Blum C., Hexemer L., Laurent S., Lang M., Pierrot T. (2024). Multi-modal transfer learning between biological foundation models. Adv. Neural Inf. Process. Syst..

[39] Saito T., Rehmsmeier M. (2015). The precision-recall plot is more informative than the ROC plot when evaluating binary classifiers on imbalanced datasets. PLoS One.

[40] Begoli E., Bhattacharya T., Kusnezov D. (2019). The need for uncertainty quantification in machine-assisted medical decision making. Nat. Mach. Intell..

[41] Hopkins A.L. (2007). Network pharmacology. Nat. Biotechnol..

[42] Meanwell N.A. (2011). Improving drug candidates by design: A focus on physicochemical properties as a means of improving compound disposition and safety. Chem. Res. Toxicol..

[43] Polishchuk P.G., Madzhidov T.I., Varnek A. (2013). Estimation of the size of drug-like chemical space based on GDB-17 data. J. Comput. Aided Mol. Des..

[44] Garey M.R., Johnson D.S. (1979).

[45] McGeoch, C., and Farré, P. (2020). The D-Wave Advantage system: An overview. Technical Report 14-1049A-A (D-Wave Systems). https://www.dwavequantum.com/media/s3qbjp3s/14-1049a-a_the_d-wave_advantage_system_an_overview.pdf.

[46] Boothby K., Bunyk P., Raymond J., Roy A. (2020). Next-generation topology of D-Wave quantum processors. arXiv.

[47] Boothby, K., King, A.D., and Raymond, J. (2021). Zephyr topology of D-Wave quantum processors. Technical Report 14-1056A-A (D-Wave Systems). https://www.dwavequantum.com/media/2uznec4s/14-1056a-a_zephyr_topology_of_d-wave_quantum_processors.pdf.

[48] D-Wave Systems (2025). Minor Embedding. https://docs.dwavequantum.com/en/latest/quantum_research/embedding_intro.html.

[49] Ou C.-H., Cheng C.-C., Chen C.-Y., Bhumkittipich K., Romphochai S. (2025). Solving optimal electric vehicle charging station placement problem using digital quantum annealing. J. Commun. Netw..

[50] Ou C.-H., Chen C.-Y., Wang C.-F., Chang C.-R. (2023). Quantum-inspired vehicle routing scheme for rebalancing in bike sharing systems. Spin.

[51] Liao P.Y., Santos J.B., Ou C.-H., Chen C.-Y., Chang C.-R. (2023). Complete the last mile: Delivery problems in quantum computing. IEEE Nanotechnol. Mag..

[52] Ou C.-H., Chen J.-Y., Cao W.-C., Chen C.-Y., Jiang Y.-L., Siangprasertkij K. (2024). Proceedings of the 2024 IEEE International Conference on Quantum Computing and Engineering (QCE).

[53] Ou C.-H., Zhu B.-Y., Huang Z.-W., Chen C.-Y., Chang C.-R., Huang W.-H. (2023). Proceedings of the 2023 IEEE International Conference on Quantum Computing and Engineering (QCE).

[54] Ou C.-H., Jiang D.-J., Chen C.-Y., Yu L.-P., Chang C.-R. (2022). Proceedings of the 2022 IEEE International Conference on Quantum Computing and Engineering (QCE).

[55] Ou C.-H., Kan Y.-W., Ohzeki M., Chen C.-Y. (2025). Proceedings of the 2025 IEEE International Conference on Quantum Computing and Engineering (QCE).

[56] Walters W.P. (2019). Virtual chemical libraries. J. Med. Chem..

[57] Bajorath J. (2001). Selected concepts and investigations in compound classification, molecular descriptor analysis, and virtual screening. J. Chem. Inf. Comput. Sci..

[58] Erlanson D.A., McDowell R.S., O’Brien T. (2004). Fragment-based drug discovery. J. Med. Chem..

[59] Ajagekar A., You F. (2023). Molecular design with automated quantum computing-based deep learning and optimization. npj Comput. Mater..

[60] Yanagisawa K., Fujie T., Takabatake K., Akiyama Y. (2024). QUBO Problem Formulation of Fragment-Based Protein–Ligand Flexible Docking. Entropy.

[61] Meuser L., Patsilinakos A., Faccioli P. (2025). De novo design of protein-binding peptides by quantum computing. J. Chem. Theory Comput..

[62] Rønnow T.F., Wang Z., Job J., Boixo S., Isakov S.V., Wecker D., Martinis J.M., Lidar D.A., Troyer M. (2014). Defining and detecting quantum speedup. Science.

[63] Quinton F.A., Myhr P.A.S., Barani M., Crespo del Granado P., Zhang H. (2025). Quantum annealing applications, challenges and limitations for optimisation problems compared to classical solvers. Sci. Rep..

[64] Kim S., Ahn S.W., Suh I.S., Dowling A.W., Lee E., Luo T. (2025). Quantum annealing for combinatorial optimization: A benchmarking study. npj Quantum Inf.

[65] Pelofske E., Hahn G., Djidjev H.N. (2023). Solving larger maximum clique problems using parallel quantum annealing. Quantum Inf. Process..

[66] King J., Yarkoni S., Raymond J., Ozfidan I., King A.D., Nevisi M.M., Hilton J.P., McGeoch C.C. (2019). Quantum annealing amid local ruggedness and global frustration. J. Phys. Soc. Jpn..

[67] Glover F., Kochenberger G., Du Y. (2019). Quantum Bridge Analytics I: A tutorial on formulating and using QUBO models. 4OR.

[68] MacLeod B.P., Parlane F.G.L., Morrissey T.D., Häse F., Roch L.M., Dettelbach K.E., Moreira R., Yunker L.P.E., Rooney M.B., Deeth J.R. (2020). Self-driving laboratory for accelerated discovery of thin-film materials. Sci. Adv..

[69] Aramon M., Rosenberg G., Valiante E., Miyazawa T., Tamura H., Katzgraber H.G. (2019). Physics-inspired optimization for quadratic unconstrained problems using a digital annealer. Front. Phys..

[70] McGeoch C.C., Wang C. (2013). Proceedings of the ACM International Conference on Computing Frontiers – CF’13.

[71] Sharma R., Katukam R., Nagulapally A. (2026). Quantum annealing for combinatorial optimization: Foundations, architectures, benchmarks, and emerging directions. arXiv.

[72] Romero S., Gupta S., Gatlin V., Chapkin R.S., Cai J.J. (2025). Quantum annealing for enhanced feature selection in single-cell RNA sequencing data analysis. Quantum Mach. Intell..

